# Urgency in the Treatment of Sudden Sensorineural Hearing Loss

**DOI:** 10.7759/cureus.40409

**Published:** 2023-06-14

**Authors:** Thomas Kepler, Shawn Flanagan, Carl Hoegerl

**Affiliations:** 1 Internal Medicine and Neurology, Liberty University College of Osteopathic Medicine, Lynchburg, USA; 2 Internal Medicine, Liberty University College of Osteopathic Medicine, Lynchburg, USA; 3 Neurology, Centra Health System, Lynchburg, USA

**Keywords:** acoustic neuroma, otolaryngology, ent emergency, hearing loss, sudden sensorineural hearing loss

## Abstract

Sudden sensorineural hearing loss (SSNHL) is considered an otolaryngologic emergency that must be treated within 72 hours. Failure to treat within that time frame typically results in permanent hearing loss. Here, we present a case of SSNHL confirmed by an audiogram that was treated as eustachian tube dysfunction. Aggressive management measures started at follow-up failed to improve the hearing loss. This case demonstrates the need for early recognition and ENT referral for SSNHL.

## Introduction

Sudden sensorineural hearing loss (SSNHL) is considered an otolaryngologic emergency with high morbidity if missed [1]. This is defined as rapid deterioration of hearing measured as SNHL over three pure tone frequencies of 30 dB (decibels) or more [2]. SSNHL often presents as unilateral hearing loss in older individuals with no precipitating factors [3]. Bilateral SSNHL (BSSNHL) is less common, more severe, and more commonly affects younger age groups [3,4]. Failure to treat within 72 hours of onset greatly diminishes the likelihood of full recovery of hearing. Family physicians must be able to promptly differentiate between conductive hearing loss and SNHL to prevent irreversible hearing loss.

The small space of the middle ear makes the study of the pathophysiology of SSNHL difficult. At this moment, little is known about the etiology of the disease. In up to 71% of cases, the cause is idiopathic and can resolve without any intervention [5]. Vascular etiology, cochlear trauma, and viral infection are three of the more prominent theories for idiopathic SSNHL [1]. Additional etiologies identified by a meta-analysis of 23 studies for SSNHL include the following: infectious disease (12.8%), otologic disease (4.7%), trauma (4.2%), hematologic (2.8%), and neoplastic (2.3%) [5]. Damage to the cochlea or hair cells is a common outcome for many of these etiologic agents [3]. Here, we discuss the common presentation of SSNHL to illustrate the importance of emergent otolaryngology (ENT) referral to family physicians. 

## Case presentation

The patient is a 45-year-old male who presented to the neurology clinic with a five-day history of gradual onset hearing loss in his left ear that began while he was on vacation in North Carolina. He reported that his left ear felt “muffled” and described his hearing as though there was a “tin can” placed over his ear. This caused him to have sounds that echoed in his hearing. He had trouble hearing out of the affected ear. He had no history of permanent hearing loss but did have a history of eustachian tube dysfunction (ETD) that caused temporary hearing loss, which was treated successfully with nasal sprays several years prior. There were no other complaints or no traumatic events prior to his decrease in hearing. On physical examination, there was a small amount of fluid noted behind the left tympanic membrane.

The patient was given a topical nasal steroid (TNS) as well as saline nasal sprays for treatment of suspected ETD with a one-week follow-up. At his follow-up appointment, his audiogram showed an approximately 10 dB difference between the left ear and the right ear at 250 Hz. Overall, the left ear showed mild-to-moderate hearing loss. The left ear had a 20 dB loss in the low frequencies and close to a 50 dB loss in the high frequencies. There was no significant hearing loss in the right ear. Word scores were significantly decreased on the left in comparison with the right. A tympanogram on the left showed a type AS (reduced peak height) tympanogram classification secondary to a peak between ±100 daPa (decaPascals) with a compliance less than 0.3 mL. The pure tone average was 22 dB on the left and normal on the right.

**Figure 1:**
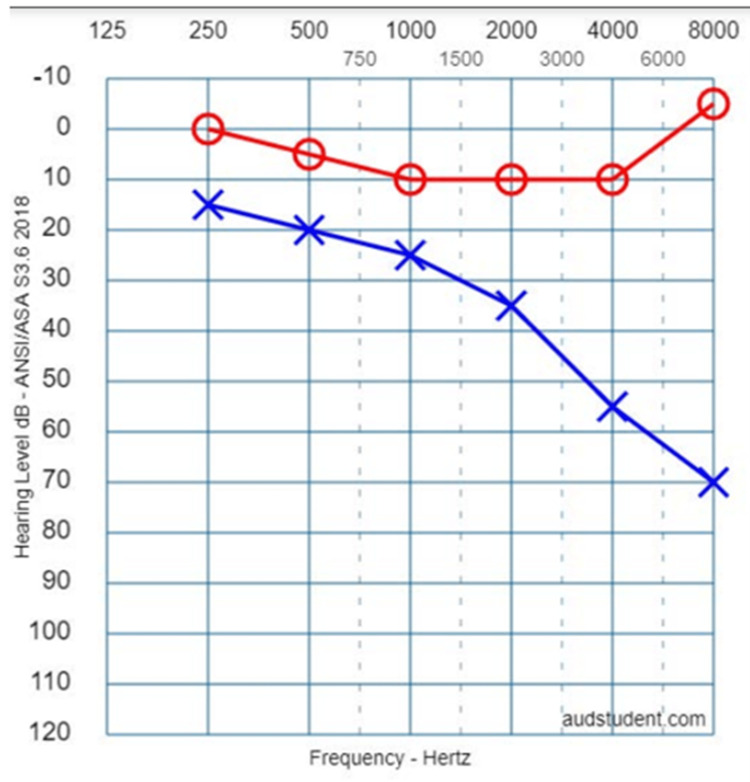
Audiogram. Audiogram recreated with permission from AudStudent.com [6]. The image reflects the original audiogram of this patient.

A repeat audiogram from several months later showed moderate-to-severe hearing loss. There was a 15 dB difference between the left ear and right ear at 250 Hz. The left ear showed a 30 dB loss in the low frequencies and an 80 dB loss in the high frequencies (Figure 1). No significant hearing loss was present in the right ear. He was subsequently treated with a course of oral prednisone without any improvement in his symptoms. MRI with internal auditory canal (IAC) protocol (MRI of internal acoustic meatus) was later obtained and ruled out acoustic neuroma.

## Discussion

In this case, ETD was suspected, and audiogram/tympanogram was obtained to confirm that diagnosis. A tympanogram revealed type A hearing on the right and AS on the left. This finding rules out ETD and otitis media due to a peak present above -100 daPa. A follow-up audiogram confirmed a significant hearing loss on the left.

The most common treatment for SSNHL is oral steroids, intratympanic steroids, or some combination of both. Intratympanic steroid injection has been documented to be effective in randomized clinical control trials (RCCT) [7]. Hyperbaric oxygen has been studied as a treatment due to the postulation of hypoxia at the organ of Corti as the underlying cause of disease [8]. Hearing aids have been shown to be effective in patients who do not recover from sudden SSNHL [9]. If hearing loss is severe, cochlear implants have been shown to be a successful next step in restoring patient's hearing.

In this case, the patient presented outside of the 72-hour window for treatment. ETD was suspected, and the patient was treated accordingly using TNS. The patient did not improve from treatment after one week, and his hearing never returned to baseline, despite delayed treatment with oral prednisone. A left hearing aid was required to improve the patient’s quality of life. This case illustrates the necessity of considering SSNHL in a patient who presents with unilateral sudden hearing loss. Otolaryngology (ENT) referral for a hearing test and oral/intratympanic steroid therapy is the best possible treatment outcome for the patient. Starting the patient on a five-day course of oral prednisone is sufficient if ENT referral within the timeframe is not feasible.

**Figure 2:**
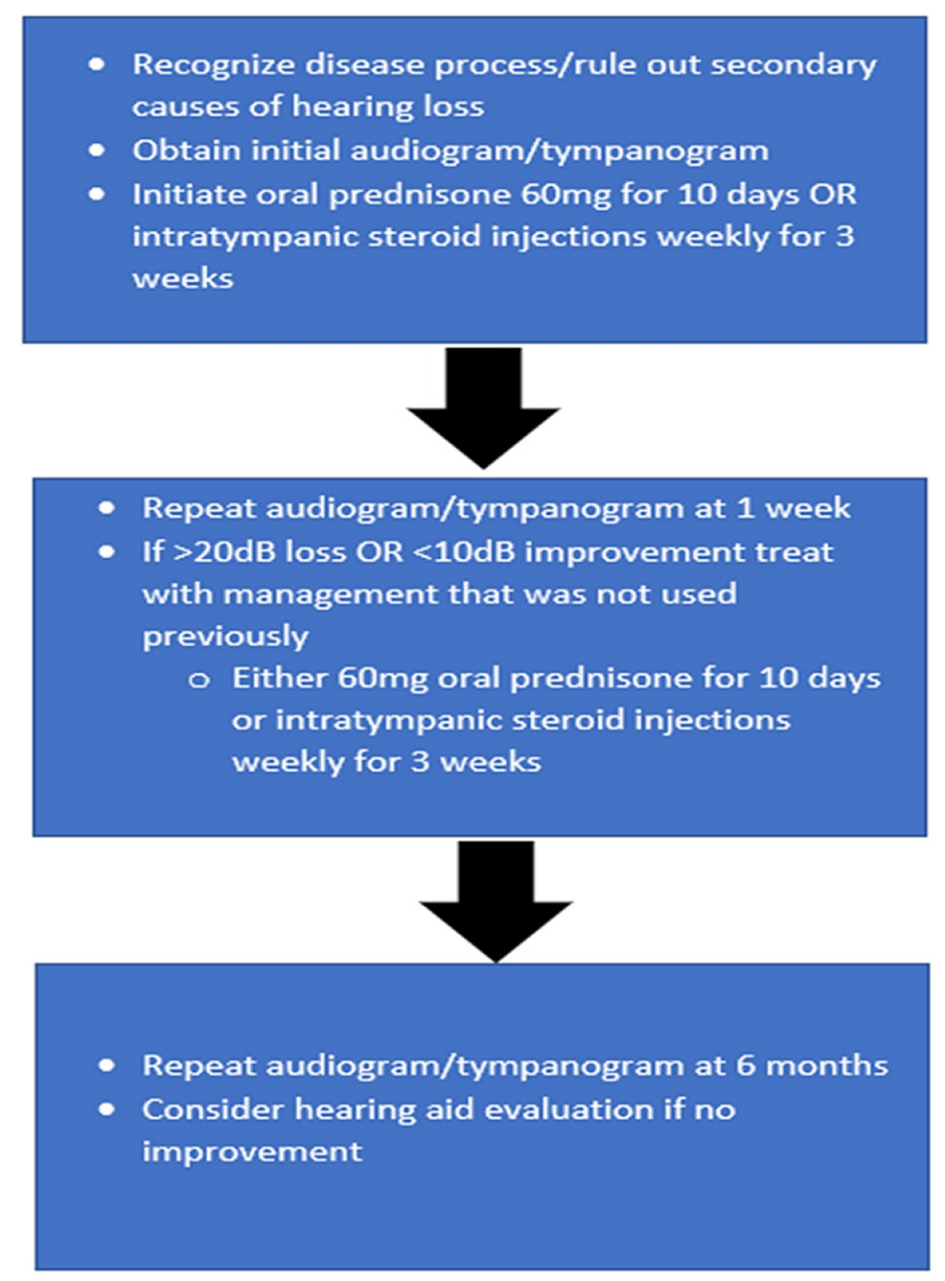
Treatment protocol.

In the treatment of SSNHL, please refer to Figure 2 for further guidance and sequence.

## Conclusions

This case helps to demonstrate the incredible need for physicians, and especially primary care providers, to not overlook the possibility of SSNHL in a patient who presents with hearing loss. The need for accurate diagnosis and quick treatment cannot be overstated. It is important to understand that hearing loss can present in a variety of ways, including tinnitus, "muffled" hearing, and even pain. Sudden hearing loss may need to be considered an ENT emergency. When a patient presents with hearing loss, all causes of hearing loss must be entertained, including SSNHL, anterior inferior cerebellar stroke, noise damage, presbycusis, otitis media, and cerumen impaction.
